# Active Versus Passive Learning in Large-Group Sessions in Medical School: A Randomized Cross-Over Trial Investigating Effects on Learning and the Feeling of Learning

**DOI:** 10.1007/s40670-024-02219-1

**Published:** 2024-11-15

**Authors:** Peter Boedeker, Tobias Schlingmann, Joshua Kailin, Ajith Nair, Cara Foldes, David Rowley, Katherine Salciccioli, Ronald Maag, Nancy Moreno, Nadia Ismail

**Affiliations:** Baylor College of Medicine, Houston, TX USA

**Keywords:** Active learning, Lecture, Large-group, Learning, Undergraduate medical education

## Abstract

**Purpose:**

The evidence base for active learning in medical education is based largely on trials with suspect internal validity. We empirically compared the learning and feeling of learning of participants when in large-group interactive sessions and passive lectures using a rigorous trial capable of providing an unambiguous assessment of effect. Further, we evaluated if there was a differential effect based on prior achievement.

**Materials and Method:**

We conducted a two-day randomized cross-over trial with 146 s-year medical students. Passive learning involved lecture-based case presentation with minimal interaction; in the large-group interactive session, students worked in teams on the same cases. Participants completed a test of learning and a feeling of learning survey. Effects were estimated using linear mixed-effects models.

**Results:**

Participants in the large-group interactive session scored 0.27 standard deviations higher on the test of learning (*p* = 0.010) than when in the passive lecture. Learners in the lower 50% of prior achievement benefited most from active learning. The feeling of learning was 0.56 standard deviations higher in the large-group interactive session (*p* < 0.001).

**Conclusions:**

Transformation of passive lectures to interactive learning sessions is feasible, has the potential to close achievement gaps by benefiting the lowest achieving learners the most, and provides students a greater sense of learning than passive lectures.

**Supplementary Information:**

The online version contains supplementary material available at 10.1007/s40670-024-02219-1.

## Introduction

Active learning is widely accepted as more effective than lecture or didactic teaching across a wide range of subject areas, including science and medicine. Beginning with Freeman’s highly cited review of undergraduate STEM (science, technology, engineering and mathematics) classes [1], the use of active learning strategies has been accepted prima facie as more effective than lecture for science teaching at a range of educational levels. However, how strong is the evidence? The strength of an evidence base depends on the internal validity of the trials that produced the evidence. A recent systematic review of the research on active learning, including those evaluations found in Freeman et al. and subsequent research in medical education, found that a majority of studies contain several internal validity issues [2]. These internal validity issues include the use of a non-random research design without an appropriate quasi-experimental method to account for pre-intervention differences, substantial differential attrition between treatment and control groups (e.g., > 25%), and different time frames of implementation (e.g., comparing one year of data in which active learning is implemented to a previous year acting as the control group). These threats to internal validity weaken a researcher’s ability to make causal claims regarding the effectiveness of active learning.

Internal validity issues are prevalent in the evaluation of specific modalities of active learning in medical education, as identified in separate reviews of medical education research. A review of the effect of a flipped classroom format showed that of the nine trials that had a control group, five used a historical control rather than a concurrent control and two compared post-treatment outcomes without accounting for pre-treatment differences [3]. More recent assessments of the flipped classroom continue to suffer from internal validity threats because authors compared one cohort of learners to a previous cohort without analytically controlling for potential confounders [4–6]. Additional reviews show a lack of randomized designs in the evaluation of problem-based learning [7] (PBL; only 16 of 124 trials used randomization) and team-based learning [8] (TBL; only 1 of 8 trials had participant randomization).

While there have been many papers published on active learning in medical education, the strength of the evidence of its effectiveness is limited. Given the numerous school or institution-level initiatives to introduce or expand active learning within the medical and health professions curricula, there is a need for further research evaluating the implementation of active learning modalities with designs that support causal inference. Here, we offer a rigorous evaluation of the effectiveness of one modality of active learning. This study was designed to address threats to internal validity by using a randomized design with equivalent groups, matched content, and matched implementation time frame.

We specifically focus on the purposeful integration of active learning into existing lectures, transforming them into large-group interactive learning sessions (LGIs) [9–11]. LGIs interweave active learning opportunities with content delivery, allowing for the immediate application of knowledge and the opportunity for an instructor to assess and provide feedback to learners in real time [12]. For example, active learning integration can occur when an instructor pauses and prompts learners to determine an appropriate treatment plan for a given case vignette that requires them to synthesize content from the current and prior lessons. The discussion of the case and critical evaluation of potential treatment options provides learners the opportunity to actively engage with the material being presented. Prior research that has evaluated a similar modification to lecture found the procedure to be beneficial; however, the outcome measure differed by group [13]. The transformation of large-group lectures into LGIs, although an arguably simpler curricular change than implementing TBL or PBL, has not received sufficient attention or evaluation in the medical education literature.

In addition, we introduce the construct “feeling of learning” to medical education from prior work in undergraduate courses [14]. While evaluation of student perception of learning has existed in various forms in medical education, the specific construct of feeling of learning is a composite of specific learner perceptions of the learning experience: enjoyment of the learning experience, perceived learning gained, effectiveness of the instructor, and desire for the same teaching modality to be used in other courses. In an undergraduate physics course, Deslauriers et al. (2019) found that feelings of learning were lower in active learning sessions compared to traditional, passive lectures, even though knowledge measured was higher following an active learning modality [14]. The authors suggest that the added cognitive load of active learning affected students’ perceptions of their learning. Feeling of learning has relevance for medical education, because learners’ perceptions about the value of an instructional approach can affect buy-in and engagement with curriculum renewal and implementation efforts.

A clear understanding of the impact of LGIs on knowledge gains and feelings of learning is needed to guide curricular design, faculty development, and communications with students and faculty in medical schools.

### Research Context and Aims

We compared the effect of participating in an LGI with a traditional lecture-based passive learning format for foundational science topics with medical students. We aimed to examine whether (1) participation in an LGI was non-inferior to traditional didactic lectures regarding student knowledge, and (2) whether participation in an LGI promoted different learning outcomes across subsets of learners based on prior achievement. Additionally, we compared medical students’ feelings of learning between the two formats.

## Materials and Methods

Baylor College of Medicine’s institutional review board approved the study. Only data from consenting students were included in our analyses. A Consolidated Standards of Reporting Trials (CONSORT) is provided as supplementary material. Data were analyzed using R (R Core Team, 2023).

### Study Design

We conducted a randomized cross-over trial with second-year medical students (MS2s) at a single U.S. institution over a two-day period. The learners had been previously exposed to TBL during their first year of coursework; however, their experience with lectures was largely in a passive format. Simple randomization was completed by the first author using Excel to produce random values for group assignment. Given that the two classrooms were at different locations, students were informed of their room assignment prior to the first day of the experiment. Each group participated in either an LGI or a large-group lecture on the first day with the other modality the second day.

Day one focused on hypertension and day two on electrocardiogram interpretation, each conducted in a 50-min session and starting simultaneously at 8 am. Each group had the same instructor for both LGI and lecture-based learning sessions. Instructors were selected for their prior experience teaching in the medical school, expertise in the subject matter, and for having completed training on the use of active learning strategies. Because of the differences in topics, we did not anticipate carryover effects. The first and second author observed the classrooms to verify that the learning modality (LGI or lecture) was implemented as intended.

The two learning conditions were developed such that the only difference between them was the active engagement of learners. The large-group lecture consisted of clinical vignette presentation with minimal interaction between the instructor and students. The active learning condition required students to work collaboratively in teams of 4 to 6 on the same clinical vignettes. Each vignette included multiple-choice questions. In the passive lecture, the instructor presented the questions and talked through each of the options for the multiple-choice questions. In the LGI, the learners were given 1 to 2 min to discuss the question and select an answer. This was followed by a brief explanation of the answer by the instructor. Between vignettes, the instructor gave a brief presentation of material that connected to the next clinical vignette. All participants, regardless of modality, were presented with identical learning objectives, PowerPoint slides, content, clinical cases, and questions. When developing the lessons, each instructor created PowerPoint slides and clinical cases for one lesson and then shared their work with the other instructor. Doing so provided consistency of the learning content and presentation materials, regardless of instructional format. At the conclusion of each session, all learners completed a feeling of learning survey and then a test of learning.

### Measures

#### Test of Learning

Three faculty members worked independently of the instructors and, without reviewing lesson materials, developed the 12-question test of learning for each topic. These items were reviewed and refined by additional team members. Only the session objectives were shared between the lesson instructors and assessment developers. By blinding the instructors to the assessments and the assessment developers to the session materials, we eliminated the possibility of bias in the developed assessments or instructors “teaching to the test.”

#### Feeling of Learning

The feeling of learning (FOL) survey was adapted from previous research [14]. Each item was Likert-scaled with 5 response options ranging from Strongly Disagree to Strongly Agree. The four items used were “I enjoyed this session on ___,” “I feel like I learned a great deal from this session,” “The instructor was very effective at teaching the material,” and “I wish all of my courses were taught in the same way as this session.” A single question was added: “I feel prepared to apply the material I learned in another class or in a future clinical context such as clerkships.”

#### Covariates

Covariates included first-year academic achievement and demographic information. The 12 course grades from the first year of medical school were averaged to produce a single score, similar to a grade point average but in the original metric of the course grades (0 to 100). This was then dichotomized using a median split, so that half of the learners are in the lower 50% of prior achievement and half of the learners are in the upper 50% of prior achievement. Additional covariates included sex (male/female), age, and race/ethnicity, with categories of Asian, Black, White (non-Hispanic/Latinx), White (Hispanic/Latinx), multiracial, and unknown. Prior evidence indicates differences among groups along lines of race/ethnicity and sex exist on medical exams [15]. By including these factors, we accounted for potential residual differences in performance.

### Analysis

#### Outcome: TOL

The sum score of the twelve items within the lesson was converted to z-scores for analysis. As z-scores, each TOL score is the deviation of the individual score from the average of scores in terms of the number of standard deviations of the distribution of scores. For example, if a participant in the LGI session for the hypertension lesson earned a z-score of 0.2 on the TOL, then this participant scored 0.2 standard deviations above the mean of scores on the hypertension TOL.

#### Outcome: FOL

To analyze FOL, composite FOL scores were created from the four FOL questions using principal components analysis (PCA). PCA is a data reduction strategy, wherein a reduced set of composite scores are created with the purpose of explaining variability in the items. The number of components determines the number of composite scores that are created for each. The number of components is determined using an eigenvalue > 1 rule of thumb, parallel analysis, and review of the component loadings of items. Components with an eigenvalue greater than 1 represent a composite that can explain more variability in the observed items than a single item and therefore would be a useful component for data reduction. In a parallel analysis, a PCA is conducted on a randomly generated dataset with the same number of items as the observed dataset, but these items are uncorrelated. Any component from the observed dataset that is greater than a component extracted from the randomly generated, uncorrelated data are considered to exist beyond chance and should be retained. Finally, the component loadings indicate the association between each item and the derived component; high loadings across items would indicate that the component is representing all items well. Because the FOL items are ordinal, the PCA is based on the polychoric correlation matrix rather than the Pearson *r* correlation matrix. The reliability of the four items is evaluated based on Cronbach’s alpha.

#### Analysis Plan

Linear mixed-effects models were used to evaluate differences in student knowledge and feeling of learning between LGI and lecture-based learning sessions. The linear mixed-effects model accounts for within-participant variability while making within-participant comparisons. The following model was fit:

$${\text{y}}_{\text{ti}} \, \text{=} \, {\text{b}}_{{0}{\text{i}}} \, \text{+} \, {\text{b}}_{1}{\text{Conten}}{\text{t}}_{\text{t}} \, \text{+} \, {\text{b}}_{2}{\text{Grou}}{\text{p}}_{\text{i}} \, \text{+} \, {\text{b}}_{3}{\text{Treatmen}}{\text{t}}_{\text{ti}} \, \text{+} \, \sum\limits_{{\text{k}}= \text{1} }^{\text{K}}{{\text{b}}}_{{\text{k}}+ \text{3} }{\text{x}}_{\text{ki}} \, \text{+} \, {\text{e}}_{\text{ti}} \, \text{+} \, {\text{u}}_{{0}{\text{i}}}$$

where ${\text{y}}_{\text{ti}}$ is the outcome observed for participant *i* at time *t*, ${\text{Conten}}{\text{t}}_{\text{t}}$ is the content of the lesson (ECG interpretation or hypertension) at time *t*, ${\text{Grou}}{\text{p}}_{\text{i}}$ is the instructional modality sequence group that participant *i* was randomized to, and ${\text{Treatmen}}{\text{t}}_{\text{it}}$ is an indicator for treatment condition at time *t* for participant *i* (LGI or lecture). The coefficient of the treatment indicator (${\text{b}}_{3}$) is the estimate of the effect of being in an LGI versus lecture. The summation captures covariates including prior achievement and demographic characteristics. The error terms, ${\text{e}}_{\text{ti}}$ and ${\text{u}}_{{0}{\text{i}}}$, capture residual variability due to within- and between-person differences in outcomes, respectively, that are unexplained by the included variables.

To evaluate if prior achievement moderated the observed effects on knowledge or feeling of learning, prior performance was dichotomized using a median split, and the impact of participation in an LGI was compared between those in the upper and lower 50% of prior achievement. Analytically, this was accomplished by interacting the median-split indicator with the treatment indicator.

Regarding the question “I feel prepared to apply the material I learned in another class or in a future clinical context such as clerkships,” Wilcoxon signed-rank tests were used to compare the responses of learners in the LGI and lecture-based conditions for each lesson.

#### Power

The necessary sample size to detect a minimum effect of interest was determined by statistical simulation. The minimum effect of interest is a standardized mean difference of 0.2, equal to one-fifth of a standard deviation in the difference between LGI and lecture-based learning conditions on the test of learning. In total, 142 participants were needed to detect this effect with power of 0.80 and alpha of 0.05. With 181 students in the MS2 course, 39 students (21.5%) could choose to not consent or participate in the experiment, and we would remain adequately powered to detect the effect of interest.

## Results

### Participant Characteristics

In total, 146 students consented and participated in both conditions (see CONSORT diagram in supplementary material). We compared consenting vs. non-consenting student demographics and prior achievement and found a statistically significant difference only in the proportions of students based on race or ethnicity (see Table 1). Individual comparisons show that differences in the proportion of learners who are Asian, White, and Unknown between groups existed. Differences in the willingness of learners to participate in an educational research study may have an implication on the generalizability of findings. Learners who are unwilling to have their data included in analyses may be more or less likely to benefit from participation in an LGI format. There were no significant differences in prior achievement or demographics based on the sequence of instructional modality (see Table 2). This lack of significant differences supports the internal validity of the design and estimated effect.

**Table 1:**
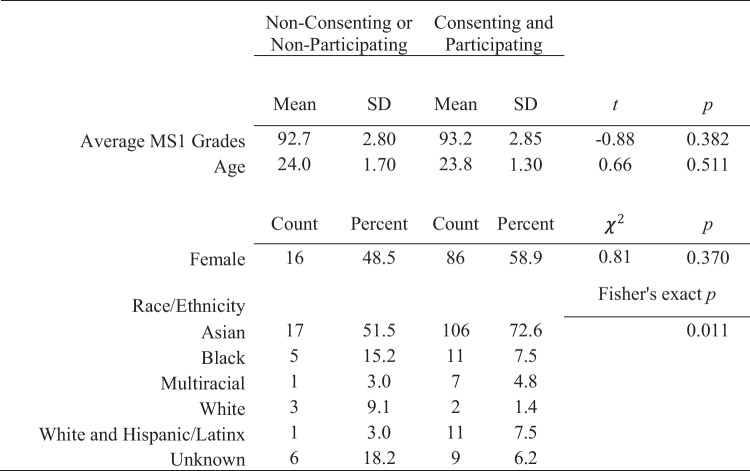
Comparison of demographics and prior performance of consenting/participating vs. non-consenting/non-participating learners

**Table 2:**
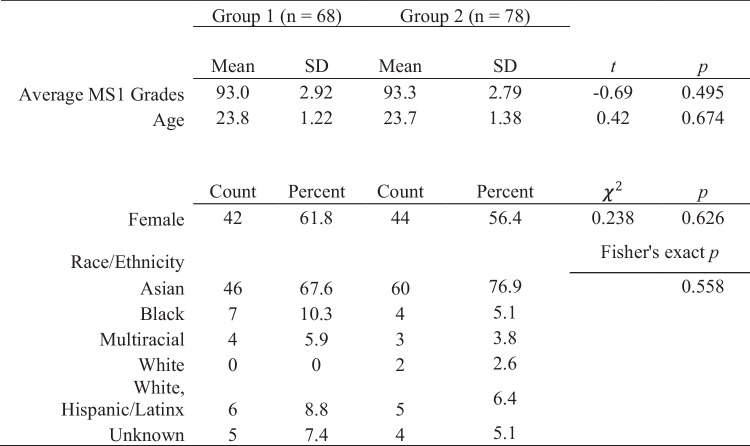
Demographics and prior performance of learners in groups 1 and 2

### Test of Learning

**Fig. 1:**
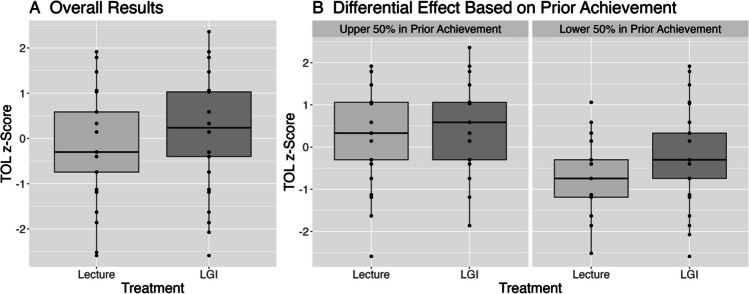
Test of learning results (**A**) overall and (**B**) by prior achievement. LGI = large-group interactive learning session

Controlling for prior achievement and demographic characteristics, learners in an LGI scored 0.27 standard deviations higher, 95% CI (0.07, 0.47), *p* = 0.010, than in the lecture-based session on the test of learning (see Table 3, TOL Main Effects). This result indicates that, overall, learners participating in the LGI were able to perform approximately a quarter of a standard deviation higher on the assessment of knowledge than when in lecture (see Fig. 1A). In the model of main effects, the only additional variable that is statistically significant is the indicator for being in the lower 50% of prior achievement (*b* = − 0.71, *p* < 0.001). This result indicates that those in the lower 50% of prior achievement scored nearly three-quarters of a standard deviation lower than their peers in the upper 50% of prior achievement.

**Table 3:** Test of learning and feeling of learning model results

	Test of learning (TOL)				Feeling of learning (FOL)			
	Main Effects		Moderation		Main Effects		Moderation	
	Estimate	*p*	Estimate	*p*	Estimate	*p*	Estimate	*p*
Intercept	0.20	0.161	**0.30**	**0.041**	− 0.18	0.219	− 0.14	0.362
Content (hyper)	0.02	0.857	0.02	0.856	− 0.18	0.074	− 0.18	0.075
Group (2)	0.09	0.412	0.09	0.412	0.04	0.732	0.04	0.732
LGI participation	**0.27**	**0.010**	0.05	0.715	0.56	** < 0.001**	**0.48**	**0.001**
Male	0.06	0.622	0.06	0.622	0.09	0.488	0.09	0.488
Black	− 0.21	0.343	− 0.21	0.343	− 0.40	0.096	− 0.40	0.096
Multiracial	− 0.25	0.363	− 0.25	0.363	0.10	0.752	0.10	0.752
Unknown	− 0.36	0.135	− 0.36	0.136	− 0.13	0.621	− 0.13	0.621
White	− 0.09	0.859	− 0.09	0.859	− 0.24	0.664	− 0.24	0.664
WHL	− 0.08	0.717	− 0.08	0.717	− 0.04	0.858	− 0.04	0.858
Age (centered)	0.09	0.055	0.09	0.055	− 0.04	0.435	− 0.04	0.435
Lower 50% prior achievement	− **0.71**	** < 0.001**	** − 0.93**	** < 0.001**	− 0.05	0.707	− 0.13	0.434
LGI participation × lower 50% prior achievement			**0.43**	**0.03**			0.16	0.434

Lower 50% prior achievement = median split of the average course grades from the first year (prior year) of medical school of learners; reference group is upper 50% of prior achievement. For Content, reference group is ECG. For Group, reference group is Group 1. *WHL*, White and Hispanic/Latinx; *LGI* large-group interactive. In bold are results with *p* values less than 0.05

The moderation analysis indicated that learners in the lower 50% of prior achievement benefited more from learning in the LGI session than those in the upper 50% of prior achievement. Learners in the lower 50% of prior achievement scored 0.43 standard deviations, 95% CI (0.04, 0.82), *p* = 0.030, higher in the LGI session than when in the passive lecture. Although the benefit of participating in the LGI session was most evident for learners in the lower 50% of prior achievement, the estimated effect of participating in an LGI remained positive for those in the upper 50% of prior achievement, though not statistically significant, *b* = 0.05, *p* = 0.715 (see Table 3, TOL Moderation and Fig. 1B). Additional control variables were not statistically significant.

### Feeling of Learning

PCA results indicated that a single component and therefore a single composite score for each person was sufficient in explaining 73% of the variance between items. Only one eigenvalue was greater than one, and the results of a parallel analysis further supported the use of a single component. Component loadings of the four items were all greater than 0.80. Reliability with the four items was 0.87. Therefore, we could proceed with analyses using a single composite score based on the four FOL items.

**Fig. 2:**
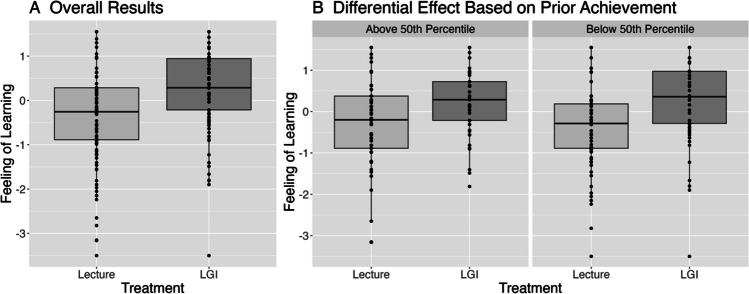
Feeling of learning results (**A**) overall and (**B**) by prior achievement. LGI = large-group interactive learning session

Participants in the LGI reported a 0.56 standard deviations higher feeling of learning, 95% CI (0.36, 0.76), *p* < 0.001, compared to those in the lecture-based learning session (see Table 3, FOL Main Effects). No additional covariates were statistically significant. Regarding the potential differential effect of LGI participation for those within the lower 50% of prior achievement, the LGI did not have a statistically significantly larger effect on feeling of learning, 0.16, 95% CI (− 0.24, 0.56), *p* = 0.434 (see Table 3, FOL Moderation). Figure 2a graphically displays the overall effect of participating in an LGI compared to a passive lecture on feeling of learning, and Fig. 2b shows the differences in feeling of learning within prior achievement groups. These differences are largely the same, confirming the non-significant moderation result.

For the question “I feel prepared to apply the material I learned in another class or in a future clinical context such as clerkships,” there was a statistically significant difference between the active learning and passive learning groups in the hypertension lesson (*W* = 3364, *p* = 0.004) but not in the electrocardiogram interpretation lesson (*W* = 2974.5, *p* = 0.188).

## Discussion

A randomized cross-over trial was conducted to compare the effect of participating in a large-group lecture and an LGI on knowledge and feeling of learning. The design of the trial allows for the estimation of the causal effect of participating in an LGI, a form of active learning, in comparison to passive lecture. The experiment took place over a two-day period with second-year medical students in a cardiology course. Scores on the test of learning and students’ feeling of learning were on average higher in the active learning modality compared to the passive learning modality. The LGI had a greater positive effect on learning for those in the lower 50% of prior achievement.

### Academic Achievement

Our findings align with the positive academic benefits found in undergraduate STEM courses [1, 16] and medical education [17]. The overall effect was found to be 0.26 standard deviations difference in favor of LGIs. For the simple instructional difference of allowing learners to discuss a vignette prior to hearing the answer, this effect is practically significant. Of particular interest is the added benefit for learners with the lowest prior achievement. Students who struggle academically in medical school have a higher likelihood of not completing their medical education [18]. Remediation can be costly in terms of time and resources for both learners and institutions; therefore, implementing strategies that support all learners during regular learning opportunities is critical. We found that students who performed in the lower 50% in the prior year of medical school benefited most from participating in an LGI, with no detriment to those whose prior performance was in the top 50%. These results align with prior findings that active learning can close achievement gaps, while benefiting all learners, and are specific to the increasingly common approach of integrating small-group active learning exercises into large-group lectures [16, 17].

In our experiment, medical case vignette review in the LGI condition involved learners working in groups. Based on a social constructivist learning theory, working collaboratively allows learners to operate within their zone of proximal development, leading to greater learning gains [19]. Furthermore, accountability to the group may motivate learners to engage with the content more deliberately. Support from peers and motivation of accountability could be contributing factors to the greater gains found in the active learning condition, with possible positive effects on sense of belonging and identity [20]. Exploring these potential mechanisms of change (e.g., feeling of peer support and motivation) and additional outcomes (e.g., sense of belonging, identity) are areas of potential future research.

### Feeling of Learning

In contrast to prior findings with undergraduates [14], medical students felt they learned more in the LGI compared to the lecture-based learning classroom. The feeling of learning items focused on enjoyment of the session, self-perceived learning, effectiveness of the instructor, and desire for all courses to be taught in this manner. Responses were higher in favor of the active learning session across all four items for both days of instruction, indicating that no single item stood out above the rest in determining this result. Further, the result was not moderated by the prior academic performance of the learners. The contrast of this finding to Deslauriers et al.’s result in which those in the active learning condition had a lower feeling of learning could possibly be because of our participants’ stage of learning or the content being taught. The students in our experiment have already completed at least bachelor’s degrees and one year of medical school. This advanced stage of learner may correlate with greater abilities to evaluate their own learning. Exploring differences in “feeling of learning” as a way of looking at learner perceptions of medical teaching innovations warrants further investigation and can complement evaluations of differences in academic outcomes.

Additionally, we found differences between lesson topics in the responses of learners to the question “I feel prepared to apply the material I learned in another class or in a future clinical context such as clerkships,” with a significant difference between LGI and lecture conditions in the hypertension lesson and a non-significant difference in the electrocardiogram lesson. The topics themselves likely play a factor in this difference in findings. Electrocardiogram is typically a more complex topic than hypertension. In this experiment, the electrocardiogram lesson covered a subset of objectives on the topic; when learners were asked to reflect on their ability to transfer their learning to another setting, they may have considered the entire topic rather than only the specific objectives of the session. Differences in feeling of learning over various topics and the reason for this could be investigated in future studies of active learning implementation.

### Implications

Transformation of lecture-based didactics into LGIs provides a robust approach to promoting success of all learners in the undergraduate medical curriculum. Unlike undergraduates, our medical school learners felt they learned more in the LGI. This information can be used to alleviate faculty concerns about learner receptivity to active learning integration and highlights the importance of attending to the principles of andragogy related to motivation and orientation to learning when building curricula and planning teaching [21]. Modifying lectures to be LGIs can support the complete integration of active learning throughout a medical school curriculum. This modification is feasible, applicable to many institutions, and, as we have shown, beneficial for learners.

### Strengths and Limitations

Strengths of the study include the rigorous implementation of a randomized trial design that allows for causal inferences to be made while controlling for known correlates of achievement in medical school. The well-powered trial and research design allows for unambiguous detection of notable effects, resulting in an internally valid effect estimate.

Limitations exist when generalizing our findings. Our sample included MS2s from one U.S. medical school over two days in a cardiology course. Future work could evaluate the effect of LGIs across different courses, numbers of sessions, and levels of learners. While only quasi-experimental designs may be feasible in many contexts, implementation of randomized trials is encouraged because of their ability to provide the strongest evidence for evaluations of causal mechanisms. In our sample, the differences between groups of participants were not significant; however, differences in race/ethnicity existed between consenting/participating and non-consenting/non-participating learners. Willingness to participate in the research study may be a confounding factor of the intervention effect that we were unable to control for. Additionally, we evaluated only immediate learning gains and effects on feeling of learning. Future research should consider the long-term implications of the LGI format on assessment such as Step 1 performance and clerkship success.

## Conclusions

Numerous studies have explored various active learning modalities, but the relatively few high-quality trials in medical education leave uncertainties about their effectiveness. Our rigorous and internally valid comparison of LGIs with traditional passive lectures demonstrates that incorporating active learning in large-group sessions offers significant academic benefits and positively influences students’ feeling of learning. When considering curriculum reform, medical school administrators are strongly encouraged to consider this integration, especially in support of those learners who have performed lower academically than their peers.

## Supplementary Information

Below is the link to the electronic supplementary material.

Supplementary file1 (DOCX 34.0 KB)

## Data Availability

Given the sensitive nature of student data we cannot make this available.

## References

[1] Freeman S, Eddy S, McDonough M, Wenderoth M. Active learning increases student performance in science, engineering, and mathematics. PNAS. 2014;3(23):8410–5. 10.1073/pnas.1319030111. 10.1073/pnas.1319030111 PMC4060654 24821756

[2] Martella AM, Martella RC, Yatcilla JK, Newson A, Shannon EN, Voorhis C. How rigorous is active learning research in stem education? An examination of key internal validity controls in intervention studies. Educ Psychol Rev. 2023;35(107):1–48.

[3] Chen F, Lui AM, Martinelli SM. A systematic review of the effectiveness of flipped classrooms in medical education. Med Educ. 2017;51(6):585–97. 28488303 10.1111/medu.13272

[4] Bansal S, Bansal M, Ahmad KA, Pandey J. Effects of a flipped classroom approach on learning outcomes of higher and lower performing medical students: a new insight. Adv Educ Res Eval. 2020;1(1):24–31.

[5] Day LJ. A gross anatomy flipped classroom effects performance, retention, and higher-level thinking in lower performing students. Anat Sci Educ. 2018;11(6):565–74. 29356452 10.1002/ase.1772

[6] El Sadik A, Al Abdulmonem W. Improvement in student performance and perceptions through a flipped anatomy classroom: shifting from passive traditional to active blended learning. Anat Sci Educ. 2021;14(4):482–90. 32881423 10.1002/ase.2015

[7] Trullàs JC, Blay C, Sarri E, Pujol R. Effectiveness of problem-based learning methodology in undergraduate medical education: a scoping review. BMC Med Educ. 2022;22(104):1–12. 35177063 10.1186/s12909-022-03154-8 PMC8851721

[8] Fatmi M, Hartling L, Hillier T, Campbell S, Oswald AE. The effectiveness of team-based learning on learning outcomes in health professions education: BEME Guide No. 30. Med Teach. 2013;35(12):e1608–24. 10.3109/0142159X.2013.849802. 24245519 10.3109/0142159X.2013.849802

[9] Graffam G. Active learning in medical education: strategies for beginning implementation. Med Teach. 2007;29(1):38–42. 10.1080/01421590601176398. 17538832 10.1080/01421590601176398

[10] McCoy L, Pettit RK, Kellar C, Morgan C. Tracking active learning in the medical school curriculum: a learning-centered approach. J Med Educ Curric Dev. 2018;21(5):1–9. 10.1177/2382120518765135 PMC5912289 29707649

[11] Steinert Y, Snell L. Interactive lecturing: strategies for increasing participation in large group sessions. Med Teach. 1999;21(1):37–42.

[12] Mazur E. Farewell, lecture? Science. 2009;323(5910):50–1. 19119207 10.1126/science.1168927

[13] Alaagib NA, Musa OA, Saeed AM. Comparison of the effectiveness of lectures based on problems and traditional lectures in physiology teaching in Sudan. BMC Med Educ. 2019;2019(19):1–8. 10.1186/s12909-019-1799-0 PMC6757398 31547817

[14] Deslauriers L, McCarty L, Miller K, Kestin G. Measuring actual learning versus feeling of learning in response to being actively engaged in the classroom. Proc Natl Acad Sci. 2019;116(39):19251–7. 10.1073/pnas.1821936116. 31484770 10.1073/pnas.1821936116 PMC6765278

[15] Rubright JD, Jodoin M, Barone MA. Examining demographics, prior academic performance, and United States Medical Licensing Examination scores. Acad Med. 2019;94(3):364–70. 30024473 10.1097/ACM.0000000000002366

[16] Theobald EJ, Hill MJ, Tran E, Agrawal S, Arroyo EN, Behling S, Chambwe N, Cintrón DL, Cooper JD, Dunster G, Grummer JA. Active learning narrows achievement gaps for underrepresented students in undergraduate science, technology, engineering, and math. Proc Natl Acad Sci. 2020;117(12):6476–83. 32152114 10.1073/pnas.1916903117 PMC7104254

[17] Koles PG, Stolfi A, Borges NJ, Nelson S, Parmelee DX. The impact of team-based learning on medical students’ academic performance. Acad Med. 2010;85(11):1739–45. 10.1097/ACM.0b013e3181f52bed. 20881827 10.1097/ACM.0b013e3181f52bed

[18] O’Neill LD, Wallstedt B, Eika B, Hartvigsen J. Factors associated with dropout in medical education: a literature review. Med Educ. 2011;45(5):440–54. 21426375 10.1111/j.1365-2923.2010.03898.x

[19] Vygotsky LS. Mind in society: the development of higher psychological processes. Cambridge, MA: Harvard University Press; 1978.

[20] Aker S, Şahin MK. The relationship between school burnout, sense of school belonging and academic achievement in preclinical medical students. Adv Health Sci Educ Theory Pract. 2022;27(4):949–63. 10.1007/s10459-022-10121-x. 35648244 10.1007/s10459-022-10121-x

[21] Taylor DCM, Hamdy H. Adult learning theories: implications for learning and teaching in medical education: AMEE Guide No. 83. Med Teach. 2013;35(11):e1561–72. 10.3109/0142159X.2013.828153. 24004029 10.3109/0142159X.2013.828153

