# Correction to: Active Versus Passive Learning in Large-Group Sessions in Medical School: A Randomized Cross-Over Trial Investigating Effects on Learning and the Feeling of Learning

**DOI:** 10.1007/s40670-025-02299-7

**Published:** 2025-02-05

**Authors:** Peter Boedeker, Tobias Schlingmann, Joshua Kailin, Ajith Nair, Cara Foldes, David Rowley, Katherine Salciccioli, Ronald Maag, Nancy Moreno, Nadia Ismail

**Affiliations:** Baylor College of Medicine, Houston, TX USA

**Correction to: Medical Science Educator**

10.1007/s40670-024-02219-1

The article “Active Versus Passive Learning in Large-Group Sessions in Medical School: A Randomized Cross-Over Trial Investigating Effects on Learning and the Feeling of Learning, was originally published electronically on the publisher’s internet portal on 15 November 2024 with error in Table 1.

The data under SD column were missing.

The original article has been corrected.

