# Supplementary material for: Active Versus Passive Learning in Large-Group Sessions in Medical School: A Randomized Cross-Over Trial Investigating Effects on Learning and the Feeling of Learning
Source: Med Sci Educ. 2024 Nov 15;35(1):459–67. doi: 10.1007/s40670-024-02219-1 (PMC11933506; doi:10.1007/s40670-024-02219-1)
Supplement: Supplementary file 1 — Supplementary file1 (DOCX 34.0 KB) [file 40670_2024_2219_MOESM1_ESM.docx]

Participant flow diagram for the submitted paper: *Active versus Passive Learning in Large-Group Sessions in Medical School: A Randomized Cross-Over Trial Investigating Effects on Learning and the Feeling of Learning*

181 Eligible, Second Year Medical Students

68 Analyzed

78 Analyzed

7 Additional participants lost to follow-up because they did not consent or provide assessment data

11 Additional participants lost to follow-up because they did not consent or provide assessment data

79 Received intervention B

85 Received intervention A

5 Lost to follow-up because they did not consent or provide assessment data

12 Lost to follow-up because they did not consent or provide assessment data

181 Randomized to sequence

91 Allocated to Sequence AB

91 Received intervention A

0 Did not receive intervention A because they did not come to class

90 Allocated to Sequence BA

90 Received intervention B

0 Did not receive intervention B because they did not come to class
